# Photocatalytic Degradation of Plastic Waste: A Mini Review

**DOI:** 10.3390/mi12080907

**Published:** 2021-07-30

**Authors:** Qian Ying Lee, Hong Li

**Affiliations:** 1 School of Mechanical and Aerospace Engineering, Nanyang Technological University, Singapore 639798, Singapore; QLEE012@e.ntu.edu.sg; 2 School of Electrical and Electronic Engineering, Nanyang Technological University, Singapore 639798, Singapore; 3 CINTRA CNRS/NTU/THALES, UMI 3288, Research Techno Plaza, Singapore 637553, Singapore

**Keywords:** plastic waste, photocatalytic degradation, photodegradation mechanisms, titanium dioxide catalyst

## Abstract

Plastic waste becomes an immediate threat to our society with ever-increasing negative impacts on our environment and health by entering our food chain. Sunlight is known to be the natural energy source that degrades plastic waste at a very slow rate. Mimicking the role of sunlight, the photocatalytic degradation process could significantly accelerate the degradation rate thanks to the photocatalyst that drastically facilitates the photochemical reactions involved in the degradation process. This mini review begins with an introduction to the chemical compositions of the common plastic waste. The mechanisms of photodegradation of polymers in general were then revisited. Afterwards, a few photocatalysts were introduced with an emphasis on titanium dioxide (TiO_2_), which is the most frequently used photocatalyst. The roles of TiO_2_ photocatalyst in the photodegradation process were then elaborated, followed by the recent advances of photocatalytic degradation of various plastic waste. Lastly, our perspectives on the future research directions of photocatalytic plastic degradation are present. Herein, the importance of catalytic photodegradation is emphasized to inspire research on developing new photocatalysts and new processes for decomposition of plastic waste, and then to increase its recycling rate particularly in the current pandemic with the ever-increasing generation of plastic waste.

## 1. Introduction

Plastic is a widely used product globally due to its versatile properties such as low weight, user-friendly designs, chemical resistance, excellent thermal stability, and outstanding electrical insulation. The resin identification code (RIC) system created by the Society of Plastic Industry (SPI) is often used to categorize the type of plastic during production and to facilitate post-consumer plastic recycling. The plastics with SPI number 1 to 7, including polyethylene terephthalate (PET), polyethylene (PE), polyvinyl chloride (PVC), polypropylene (PP), polystyrene (PS), etc., are present in Table 1 [1].

The global plastic production was about 2 million tons in the year 1950, which is trivial compared to the annual production nowadays (368 million tons as of 2019) [2]. According to data from Plastic Europe, the most produced plastic type is polyolefin including high-density polyethylene (HDPE), low-density polyethylene (LDPE), and PP, which are mainly used in the packaging industry. Many of those plastics produced in 1950 still exist as plastic waste in the environment. One of the reasons for the increasing global plastic production over the years in various industries is the benefit of one-time use, and thus is very convenient and cost-effective. As a result, the global plastic market size is expected to reach USD 722.6 billion by 2027 [3]. In addition, the recent COVID-19 outbreak worldwide has caused a surge in the demand for medical personal protective equipment (PPEs) such as single-use disposable face masks, medical face shields, and gloves, most of which are made of polymer materials [4]. For instance, a single-use disposable face mask consists of three layers: An inner layer (made of fibrous material), a middle layer (melt gusted filter part), and an outer layer (nonwoven) [5]. The middle filtering layer of the mask can be nanofibers and/or microfibers, which is manufactured using raw material such as PP. The outer layer of the mask can be made of polyethylene terephthalate (PET). The demand for disposable plastic products including food containers, plastic bags, and poly mailbags in the packaging industry which are made from plastic has also been growing rapidly since more people are staying at home, calling for food delivery, and online shopping particularly in the current pandemic [4].

In 2020 alone, about 52 billion single-use disposable face masks have been produced owing to the pandemic, and about 1.6 billion (3%) of them enter the oceans that need 40–50 centuries for complete degradation. Tons of microplastics will be released in the oceans during the natural degradation of these masks, which will likely enter our food chain. Moreover, other plastic wastes have also caused a variety of environmental issues, directly imposing an immediate threat to our health and survival. As a result, excessive use of plastic has caused a flood of plastic wastes entering the natural environment, bringing adverse effects to humans and the environment [6]. On top of that, more environmental issues have emerged, especially when there is a lack of proper management and handling of plastic wastes.

Recycling is one of the promising methods to reduce the detrimental effects caused by plastic wastes. PET and HDPE show a recovery rate of 19.5% and 10%, respectively, as depicted in Table 1. However, plastic such as PVC, LDPE, PP, and PS that has a recovery rate of less than 5%, depending on their applications, are barely recyclable. PVC has a recovery rate of 0%, suggesting that it is completely non-recyclable which is mainly due to the high chlorine content in its raw material and the high level of hazardous additives in it. One can see that only less than 10% of the plastic wastes are recycled on average, leaving more than 80% to accumulate in the natural environment [7].

Therefore, engineering plastic degradation processes such as photodegradation [6,8], thermo-oxidative degradation [9,10], and biodegradation [11] have attracted intensive research interest. Photodegradation has attracted our attention as it strikes an excellent balance of energy consumption, time consumption, and cost-effectiveness, holding great promise to end plastic wastes. Most importantly, it can capitalize on abundant and renewable solar energy.

**Figure 1:**
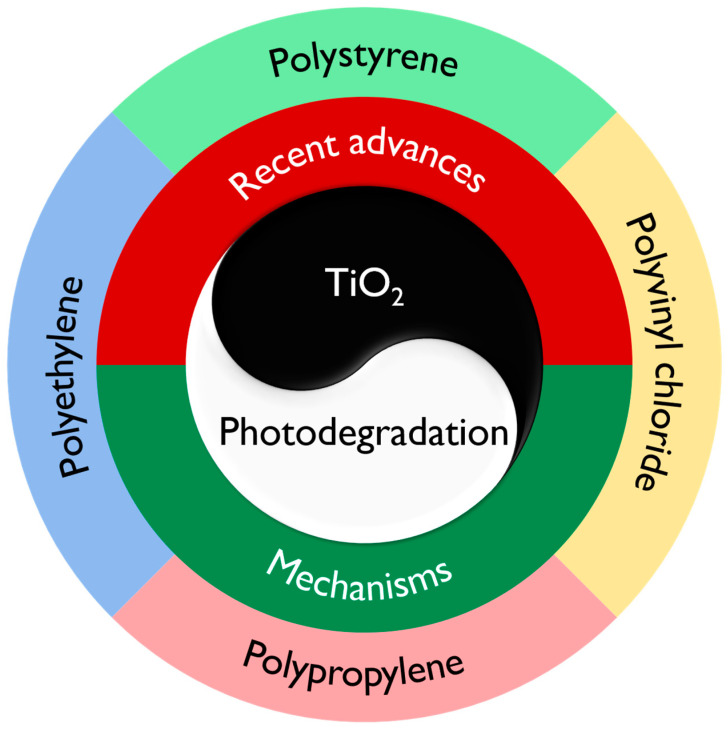
Focus of this review on catalytic photodegradation for various types of plastic polymers.

Herein, we revisit the concept of catalytic photodegradation of plastic wastes. We start with the discussion of the mechanisms involved in photocatalytic degradation of polymer, followed by summarizing the recent advances of catalytic photodegradation of various types of plastic polymers to shed light on the degradation of the entire spectrum of plastic waste, as illustrated in Figure 1. Plastic polymers including PE (LDPE, HDPE), PP, PS, and PVC are described in our review as they constitute the world’s most common plastic and most of them are challenging to be recycled. Lastly, we present our prospective on the future research directions on photocatalytic degradation of plastic wastes. 

## 2. Photodegradation of Polymers

### 2.1. Background of Photodegradation

The sun emits energy over a broad range of wavelengths from ultraviolet spectrum (UV), visible spectrum (Vis-), to infrared spectrum (IR) with decreasing energy. Ultraviolet radiation consists of electromagnetic waves with a wavelength between 100 and 400 nm [12], which is further divided into three major sub-regions. With the wavelength ranging from 100 to 280 nm, UV-C is fully absorbed by the ozone in the atmosphere. UV-B has a wavelength from 280 to 315 nm, whereas UV-A has a wavelength from 315 to 400 nm. Most of the UV-B is also absorbed by the atmosphere, while UV-A is completely reachable to the Earth’s surface. Visible light is the section of the electromagnetic spectrum that is visible to humans, covering the range of wavelength from 400 to 700 nm. Infrared radiation consists of electromagnetic waves with a wavelength from 700 to 1 mm.

UV radiation is known as the most damaging source to polymers owing to its high energy. Hence, polymers that are continuously exposed to UV radiation will undergo deterioration in their physical and chemical structure, resulting in photodegradation. Photodegradation of polymer includes chain scission, alteration of molecule’s shape, reduction in molecule’s weight, and deterioration of polymer properties typically in the presence of UV radiation and oxygen [8].

### 2.2. Photodegradation Mechanisms

There are two well-regarded photodegradation mechanisms, i.e., singlet oxygen induced oxidation [8,13] and free radical caused oxidation [14], as detailed in the following sections.

#### 2.2.1. Singlet Oxygen Mechanism of Oxidation

Singlet oxygen mechanism of oxidation involves the direct reaction of singlet oxygen with polymer. The singlet oxygen is produced due to the quenching of the excited triplet state of suitable sensitizers (^3^S), as depicted in Equation (1).

(1) S3+O32→S1+O12

**Figure 2:**
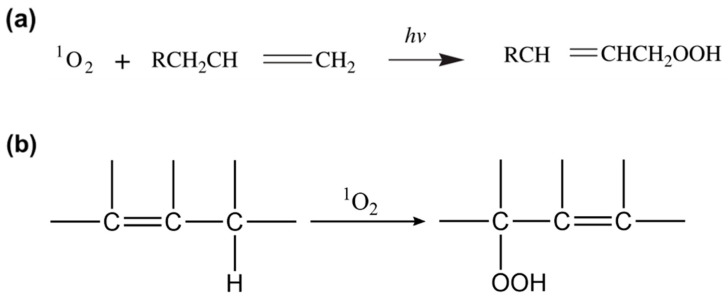
Singlet oxygen mechanism of oxidation. (**a**) Singlet oxygen oxidation of a vinyl group to form the hydroperoxide functional group (ROOH) [13]; reprinted from [13] with permission from Elsevier. (**b**) Singlet oxygen oxidation of an olefin group to form the hydroperoxide functional group (ROOH) [8].

A singlet oxygen could react with the product of a Norrish reaction, which is a photochemical reaction taking place with ketones and aldehydes. A Norrish reaction can be subdivided into type I and type II reactions, and Norrish Type II reaction causes intermolecular rearrangement of the carbonyl group (C=O) to form the vinyl group (-CH=CH_2_). Thereafter, a generated singlet oxygen that reacts with the vinyl group could then further decompose the molecule, leading to chain scission and formation of hydroperoxides functional group (ROOH), as shown in Figure 2a. Likewise, the singlet oxygen produced also leads to the formation of ROOH by the oxidation of an olefin containing allylic hydrogen, as depicted in Figure 2b.

#### 2.2.2. Free Radical Mechanism of Oxidation

**Figure 3:**
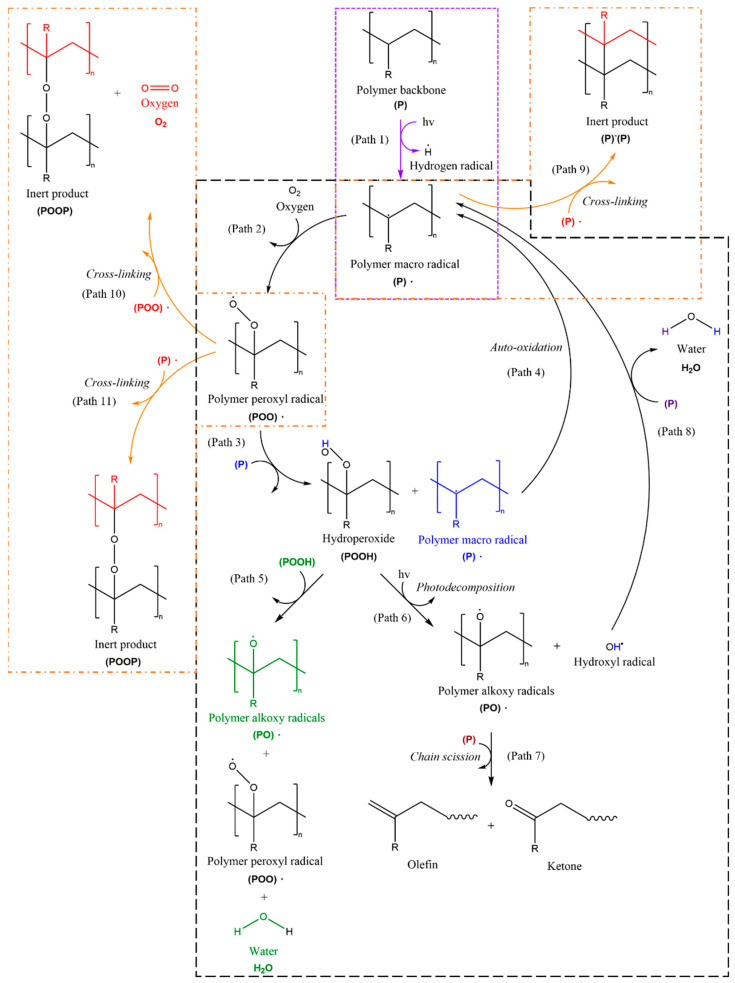
Photodegradation mechanism of polymer involving free radical oxidation. Purple dotted box: Initiation step; black dashed boxes: Propagation step; orange dash-dotted boxes: Termination step.

The free radical mechanism of oxidation involves producing free radicals that react with the oxygen. The high energy in the UV radiation breaks the C-C and C-H bonds in polymers to create free radicals. The free radicals then react with the oxygen to create the hydroxyl group (O-H) and carbonyl group (C=O). The mechanism involves three main steps, i.e., initiation, propagation, and termination, as summarized in Figure 3.

In the initiation step, the existence of chromophore group, which absorbs the incident light due to energy matching, in the polymer’s structure is important to act as the photo-initiator [8]. As a result, the absorbed light-excited charge carriers break the chemical bonds in the polymer chains to produce hydrogen radicals and polymer radicals (Path 1). In principle, non-absorbing polymers such as polyethylene (PE) and polypropylene (PP) are almost perfectly stable on exposure to solar radiation with a wavelength greater than 290 nm, since the structure contains only bonds of C-C and C-H [15]. Moreover, they have no unsaturated chromophores that can absorb light to form free radicals. However, the impurities or structural defects in the polymers, as well as carbonyl groups within the backbone of the polymer can act as chromophores to carry out the photo-initiation [6].

The potential sources for producing free radicals are carbonyl groups such as ketones and hydroperoxides. Hence, different initiation steps have been undertaken for various conditions in different polymers to form the free radicals. Those reactions can be initiated by physical factors such as direct UV initiated photolysis of C-C and C-H bond or by chemical factors such as the residues of catalyst used, incorporation of carbonyl groups, and introduction of peroxides or unsaturation site [13]. The free radicals produced can extract hydrogen atoms from the other polymers and thus initiate the photodegradation activity.

In the propagation step [8,16], polymer macro radicals formed through photo-initiation react with the oxygen to form polymer peroxyl radicals (Path 2). Subsequent reactions of polymer peroxyl radicals with another polymer produce hydroperoxides and polymer macro radicals (Path 3). Afterwards, the polymer macro radicals produced undergo auto-oxidation to repeat the formation of polymer peroxyl radicals (Path 4). In addition, the hydroperoxides react with another hydroperoxide to form polymer alkoxy radicals, polymer peroxide radicals, and water (Path 5). Moreover, the photodecomposition of hydroperoxides leads to the formation of polymer alkoxy radicals and hydroxyl radicals (Path 6). Propagation ultimately leads to chain scission to form an oxygen-containing functional group including olefin and ketone (Path 7). The hydroxyl radicals produced will extract hydrogen from another polymer to form polymer macro radicals and water (Path 8).

Lastly, in the termination step [8], the free radicals produced react with each other to undergo the crosslinking reaction for the formation of inert products (Path 9). When the oxygen pressure is high, polymer peroxyl radicals react with themselves to form inert products and oxygen (Path 10). In contrast, when sufficient oxygen cannot be maintained, polymer peroxyl radicals react with polymer macro radicals (Path 11). Conclusively, olefins and ketones are the expected products of the termination reactions [16]. The whole process causes a reduction in the molecule’s weight, and the polymer becomes more brittle, leading to further photodegradation.

## 3. Photocatalytic Degradation

In photocatalytic degradation, which is a photochemical reaction process with the help of photocatalysts, a semiconductor is often used to absorb light and to accelerate the photoreaction rate [17]. Photocatalysis is used in many applications such as removal of pollutants and bacteria [18], energy conversion [19], and water splitting for green hydrogen generation [20]. An ideal photocatalyst should be able to absorb light at room temperature, and have high stability towards photo corrosion, as well as non-toxicity.

### 3.1. Titanium Dioxide (TiO_2_) as Photocatalyst

**Figure 4:**
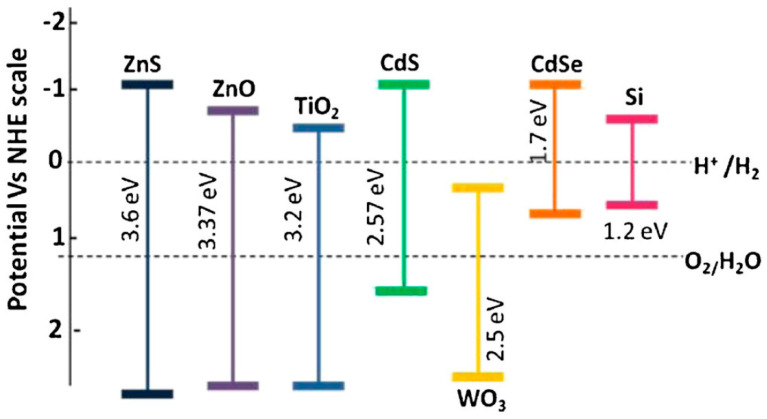
Band gap potential diagram of the most common semiconductors, used as photocatalysts at NHE scale [22]; reproduced from [22] with permission from the Springer Nature.

TiO_2_ is the most widely used photocatalyst due to its versatile properties such as high level of oxidation-reduction ability [21,22], chemical stability [21], high-temperature stability, cost-effectiveness, and environmental friendliness [23,24]. The oxidation-reduction ability of a photocatalyst depends on its energy band position w.r.t. redox potentials. As illustrated in Figure 4, using water splitting redox potentials as the reference, TiO_2_ has a more positive electrochemical potential with respect to the normal hydrogen electrode (NHE) potential. Having the valance band maximum (VBM) more positive than 1.23 eV, showing that the oxidation ability is sufficient in oxidizing water. The lower the position of VBM of a semiconductor, the higher the oxidation capability it has. In contrast, the conduction band minimum (CBM) should be more negative than the hydrogen reduction potential for affecting the reduction of water. The higher the position of CBM of a semiconductor, the higher the reduction capability it has. Thus, TiO_2_ has one of the highest oxidation capabilities among all of the semiconductors listed in Figure 4. However, one should decrease the band gap to increase light adsorption. For instance, ZnS has a too large band gap to absorb light efficiently though it has high oxidation and reduction capability. Put together, TiO_2_ has a great balance between oxidation-reduction capability and band gap size, resulting in an excellent photocatalyst.

**Figure 5:**
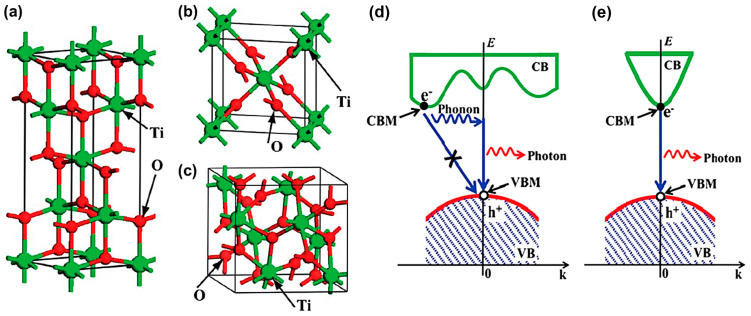
Atomic structure of (**a**) anatase TiO_2_, (**b**) rutile TiO_2_, and (**c**) brookite TiO_2_. Green and red balls represent Ti and O atoms, respectively. Schematic carrier recombination process in (**d**) anatase and (**e**) rutile TiO_2_. The e^−^ and h^+^ represent the photogenerated electron and hole, respectively. Reproduced from [27] with permission from the PCCP Owner Societies.

TiO_2_ has three crystalline forms: Anatase, rutile, and brookite [25,26,27], as depicted in Figure 5a–c. Different phases have different symmetries of the octahedral-shaped TiO_6_ fundamental building blocks [28]. Anatase and rutile forms are commonly used in photocatalytic degradation, while brookite is uncommonly used as a photocatalyst due to its unstable structure. Anatase can be converted into rutile by heating above 700 °C [25]. TiO_2_ can appear in various geometry including nanoparticles [29], nanowires [30], nanotubes [31], and other nanostructures [32].

Anatase TiO_2_ has a CBM at the energy level of E_CB_ = −0.51 V, while rutile TiO_2_ has CBM at E_CB_ = −0.31 V [33]. In contrast, their VBMs are similar at an energy level of E_VB_ = +2.69 V. Consequently, the anatase form has a slightly larger bandgap E_g_ = 3.2 V than that of rutile (E_g_ = 3.0 V), leading to a higher energy of photogenerated charges. As a result, anatase has a relatively higher photocatalytic activity, however, a relatively narrower absorption bandwidth. Moreover, anatase exhibits an indirect bandgap, while rutile and brookite show direct bandgaps, as illustrated in Figure 5d,e. Hence, the lifetime of photogenerated electron-hole pair in anatase is longer since a direct recombination of electron-hole pair is forbidden theoretically.

**Figure 6:**
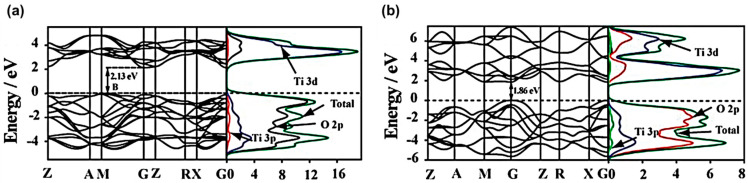
Energy band structure (left panel) and DOS (right panel) for (**a**) anatase and (**b**) rutile TiO_2_ [27]; reproduced from [27] with permission from the PCCP Owner Societies.

The detailed energy band structure and density of state (DOS) of anatase and rutile TiO_2_ are similar, as shown in Figure 6 [27]. Owing to strong O 2p-Ti 3d hybridizations, the valance bands contain O 2p and some Ti 3d states. Such strong p-d hybridizations broaden the valance bands and enhance the photo carrier transfer. In contrast, the conduction bands consist of primarily Ti 3d states with minor O 2p and Ti 3p states. It is worth noting that the calculated band gaps of anatase and rutile TiO_2_ are smaller than the measured ones due to the limitation of basic density functional theory calculations [34,35]. However, this deficiency can be corrected by the GGA+U approach with more appropriate determined U parameter values [36]. The GGA+U approach is improved in terms of the energy gap of material and impurity state in the gap. It yields an energetic sequence consistent with the experiments, E_rutile_ < E_anatase_, for 5 < U < 8 eV.

Moreover, the effective mass of photogenerated charge carriers can be obtained by parabolic fitting to the calculated CBM and VBM along a specific direction in the reciprocal space. Notably, the average effective mass of photogenerated charge carriers in anatase phase is lower than those in rutile and brookite phases, resulting in faster migration of the photogenerated electron and holes to the surface of TiO_2_ [27]. Therefore, more photogenerated charge carriers in anatase can participate in the surface reactions. Notably, the photocatalytic activity also greatly depends on other factors such as the geometry [32], the substrates [33], and the oxygen vacancy/defects density [37].

### 3.2. Other Photocatalysts

**Figure 7:**
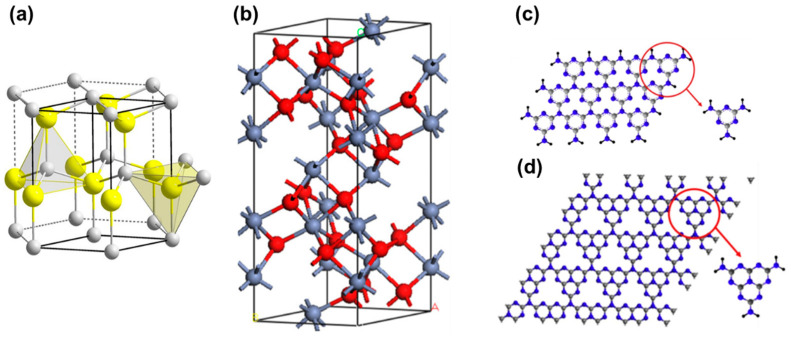
Atomic structure of typical photocatalysts for photodegradation. (**a**) ZnO wurtzite cell, Zn atoms in yellow, O atoms in grey [51]. (**b**) The hexagonal representation of unite cell of ferric oxide (hematite) with Fe atoms in blue and O atoms in red [52]; reprinted from [52] with permission from IOP Publishing. Structure of (**c**) s-triazine and (**d**) tri-s-triazine as the primary building blocks of graphitic carbon nitride with C atoms in grey and N atoms in blue [50]; reprinted from [50] with permission from Elsevier.

Zinc oxide (ZnO) having a comparable bandgap to that of TiO_2_ is often used as an alternative to TiO_2_, as presented in Figure 7a. Many reports have focused on the use of ZnO as the photocatalyst, and some results have also shown a high photocatalytic degradation rate [38,39,40,41,42]. Iron oxide [43], cadmium sulphide [44], zinc sulphide [45], tungsten oxide [46], tin oxide [47], bismuth vanadate [48], and non-metallic carbon nitride [49,50] are also often employed as the photocatalysts for photodegradation too, as depicted in Figure 7b–d. Though the photocatalyst plays the vital role in photodegradation, the degradation rate also greatly depends on the polymer structure, photocatalyst mass loading, as well as the experimental setup.

### 3.3. Photocatalytic Degradation Mechanism

When the photocatalyst such as TiO_2_ is exposed to the UV light with irradiation energy that is equal to or more than its bandgap, the electrons (e^−^) are excited from its VB to CB, creating an energetic electron (e^−^)-hole (h^+^) pair, as depicted in Equation (2).

(2) $${\mathrm{TiO}}_{2}\overset{\mathrm{hv}}{\to }{\;e}^{-}+h^{+}$$

Part of the electrons will recombine with holes swiftly in femtoseconds, and the rest has a longer lifetime [24]. The recombination of electrons-holes reduces the number of photogenerated electrons and holes transported to the TiO_2_ surface for oxidation-reduction chemical reactions. Therefore, the presence of scavengers and incorporation of trap sites, which help reduce the recombination of electrons and holes in the bulk of semiconductor, are attractive strategies to increase the catalytic activity [25]. Thereafter, the electrons and holes transferred to the surface of the semiconductor undergo interfacial charge-transfers to carry out the oxidation-reduction chemical reaction as follows.

In the reductive reaction, electrons in the CB of photocatalyst react with oxygen to produce superoxide, as illustrated in Equation (3). Then, the superoxide will undergo further reduction to produce hydrogen peroxides, which may generate hydroxides and hydroxyl radicals through the reaction with the electrons, as shown in Equation (4) [33].

(3) $$O_{2}+{\;e}^{-}\to O_{2}^{-}$$

(4) $$H_{2}O_{2}+{\;e}^{-}\to {\mathrm{OH}}^{-}+\cdot \mathrm{OH}$$

In the oxidative reaction, holes in the VB of photocatalyst react with the water in the air to form proton and hydroxyl radicals, as depicted in Equation (5). Consequently, hydroxyl radicals will decompose organic pollutants into the water and carbon dioxide. Hence, hydroxyl radical is known as the essential element in photocatalysis. Moreover, it is regarded that the interfacial charge transfer at the surface of the semiconductor is important in producing hydroxyl radicals, affecting the overall photocatalytic degradation rate [33].

(5) $$H_{2}O+{\;h}^{+}\to H^{+}+\cdot \mathrm{OH}$$

Since UV light only represents 5–8% of the solar spectrum (at sea level), it limits the efficiency of photocatalytic degradation for most photocatalysts such as TiO_2_ and ZnO. Hence, photocatalytic degradation under visible light is crucial to utilize more solar energy. To increase the photocatalytic degradation activity under visible light, the energy band gap needs to be reduced. This can be achieved by introducing structural imperfections into the crystal, a process known as chemical doping. This is possible by varying the synthesis conditions and/or adding controlled amounts of impurities such as C, N, Fe^3+^, Cr^3+^, etc. [53].

## 4. TiO_2_-Based Photocatalyst in Plastic Degradation

### 4.1. Polystyrene (PS)

**Figure 8:**
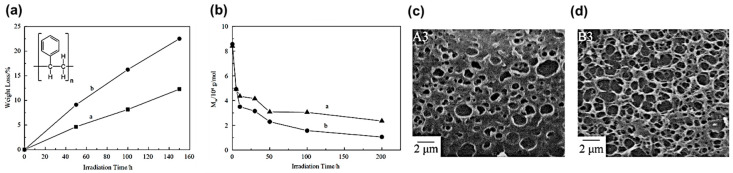
Photodegradation of PS-TiO_2_ film. (**a**) Weight loss comparison between pure PS (curve a) and PS-TiO_2_ samples (curve b) over the UV irradiation time. Inset: Chemical formula of PS. (**b**) The variations of average molecular weight M_w_ of pure PS (curve a) and PS-TiO_2_ samples (curve b) over the UV irradiation time. SEM images of (**c**) pure PS sample (A3), and (**d**) PS-TiO_2_ sample (B3) after UV irradiation for 40 h [54]; reprinted from [54] with permission from Elsevier.

The photocatalytic degradation of PS plastic [(C_8_H_8_)_n_, as shown in the inset of Figure 8a] with TiO_2_ as photocatalyst was investigated in the ambient air under UV irradiation [54]. The results showed that the degradation of PS-TiO_2_ composite exists at a much higher weight loss rate (Figure 8a), lower average molecular weight (Figure 8b), and more voids generated (Figure 8c,d), as compared to that of pure PS film degradation under UV irradiation.

**Figure 9:**
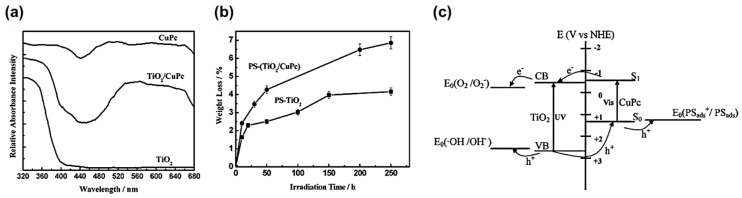
Photodegradation of PS-(TiO_2_/CuPc) film. (**a**) The UV-VIS absorption spectra of TiO_2_, TiO_2_/CuPc, and CuPc samples. (**b**) Weight loss curve of PS-TiO_2_ and PS-(TiO_2_/CuPc) samples. (**c**) The schematic charge separation mechanism of TiO_2_/CuPc sample under visible and UV light radiation [55]; reprinted (adapted) from [55] with permission from the American Chemical Society.

Various modified TiO_2_ catalysts have been employed for the photocatalytic degradation of PS including copper phthalocyanine (CuPc) sensitized TiO_2_ [PS-(TiO_2_/CuPc] [55], grated TiO_2_ (PS-G-TiO_2_) [56,57], iron phthalocyanine (FePc) sensitized TiO_2_ (PS-FePc-TiO_2_) [58], and hindered amine modified aromatic polyamide dendrimer/ polystyrene-grafted TiO_2_ (PS-HADPG-TiO_2_) [59]. From the experimental results, modified TiO_2_ photocatalysts showed a much higher photocatalytic degradation efficiency than pure TiO_2_. When the photocatalytic degradation of PS plastic was carried out with FePc-TiO_2_, HADPG-TiO_2_ or TiO_2_/CuPc as photocatalyst, it was found that these modified TiO_2_ had a widened absorption under visible light. For instance, the UV-VIS absorption spectrum of TiO_2_/CuPc shows a broad absorption peak in the visible range of 500 to 650 nm thanks to CuPc, as depicted in Figure 9a. This enhanced the ability for PS degradation under visible light, subsequently increasing the photocatalytic degradation efficiency under solar irradiation (Figure 9b).

As illustrated in Figure 9c, the UV portion of the incident light (below 388 nm) is absorbed by TiO_2_, while the visible portion of the incident light (below 685 nm) is absorbed by CuPc. Upon illumination in the visible range, an electron in CuPc is excited to a singlet state (S_1_), leaving a hole in the ground state (S_0_). As the energy level of S_1_ (−0.63 V vs. NHE) is higher than that of TiO_2_ (−0.5 V vs. NHE), the excited electron will be swept to TiO_2_ by the built-in electric field at the interface of TiO_2_ and CuPc. As such, the photogenerated electron and hole are separated and then utilized to reduce oxygen and to oxidize PS molecule, respectively. Notably, the anatase crystalline form of TiO_2_ shows higher activity than rutile TiO_2_ for photocatalytic degradation, owing to the lower recombination rate and longer lifetime, as discussed in the previous section [58,59].

### 4.2. Polyvinyl Chloride (PVC)

**Figure 10:**
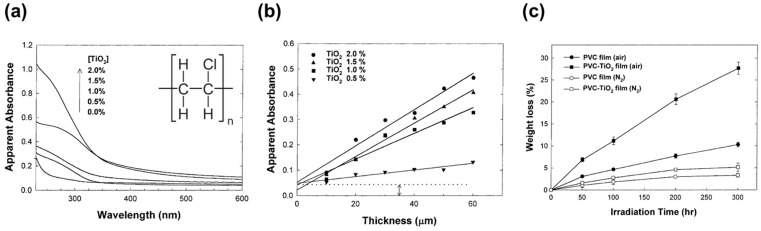
Photodegradation of PVC–TiO_2_ film. (**a**) UV–VIS absorption spectra of the PVC–TiO_2_ composite films with varying TiO_2_ contents. Inset: Chemical formula of PVC. (**b**) Apparent absorbance of the composite films at λ = 350 nm as a function of the film thickness. (**c**) Weight loss of the pure PVC and PVC-TiO_2_ (1.5 wt%) composite film during irradiation in air (filled symbols) or N_2_ (open symbols) atmosphere [60]; reprinted from [60] with permission from Elsevier.

Investigations have been also carried out to determine the photocatalytic degradation efficiency of pure PVC [(C_2_H_3_Cl)_n_, as depicted in the inset of Figure 10a], and PVC film with TiO_2_ or modified TiO_2_ as photocatalyst. As expected, the degradation efficiency is always relatively higher for the latter. According to Cho and Choi [60], light penetration into the PVC composite film depends on the concentration and size of TiO_2_, as well as the film thickness. The higher the amount of TiO_2_, the greater the light absorbance of the PVC composite film is, as displayed in Figure 10a. However, the optical transparency decreases with more TiO_2_, and thus shallower light penetration depth, as shown in Figure 10b. Moreover, the weight loss percentage is higher in air ambient compared to that in nitrogen ambient (Figure 10c). It suggests that the presence of oxygen in the air was essential for the efficient photocatalytic degradation of the polymer, agreeing with the reaction mechanism discussed in Equation (3).

**Figure 11:**
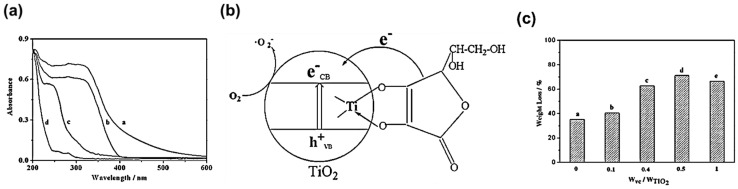
Photodegradation of PVC-VC-TiO_2_ film. (**a**) UV–VIS absorption spectra of different films before irradiation: PVC–VC–TiO_2_ film with 2 wt% TiO_2_ and 1 wt% VC (curve a), PVC–TiO_2_ film with 2 wt% TiO_2_ (curve b), PVC–VC film with 2 wt% VC (curve c), and pure PVC film (curve d). (**b**) The proposed mechanism of photocatalysis process for VC modified TiO_2_. (**c**) The weight loss rate at different mass ratios of VC to TiO_2_ in PVC-VC-TiO_2_ film; [61] reprinted from [61] with permission from Elsevier.

Interestingly, photocatalytic degradation of PVC film with the vitamin-C (VC)-modified TiO_2_ photocatalyst was investigated [61]. The specific binding of VC to the TiO_2_ surface was attributed to the fact that the Ti ions can easily form complexes with oxygen-containing ligands. The UV-VIS spectrum shows that the absorption range of PVC-VC-TiO_2_ has significantly broadened compared to that of PVC-TiO_2_ and PVC-VC, as depicted in Figure 11a. The broadened absorption was ascribed to TiO_2_ and the unsaturated bonds of VC. The redshift of the absorption threshold wavelength up to 600 nm was explained by the bidentate binding of α-substitute surface modifiers, leading to the five-membered ring at the surface of Ti atoms, as illustrated in Figure 11b. It was found that the photocatalytic activity was greatly enhanced by the formation of Ti^IV^-VC charge-transfer complex having a five-member chelate ring structure. Moreover, the optimum mass ratio of VC to TiO_2_ is 0.5 for the highest efficiency of photocatalytic activity, as shown in Figure 11c.

Upon illumination, the Ti^IV^-VC charge-transfer complex promoted the synergetic effect between VC and TiO_2_, as illustrated in Figure 11b. The photogenerated electrons are transferred from VC to the conduction band of TiO_2_ to form the superoxide radicals, according to Equation (3). The superoxide radicals attack the polymer chains nearby, which accelerate the PVC degradation via one-electron reduction of surface oxygen.

As the photocatalytic activity of TiO_2_ photocatalyst has low efficiency under visible light [62], a perchlorinated iron (II) phthalocyanine modified TiO_2_ (FePcCl_16_-TiO_2_) was developed and showed greatly improved absorption ability of visible light [63]. Similarly, the photocatalytic degradation of PVC with bismuth oxyiodide-modified TiO_2_ (PVC-BiOI/TiO_2_) [64], polyoxometalate-modified TiO_2_ (PVC-POM/TiO_2_) [65], and nano-graphite-doped TiO_2_ [PVC-(Nano-G/TiO_2_)] [66] also showed improved visible light activity due to their broadened absorption spectra. The modified photocatalysts can effectively improve the migration and separation of TiO_2_ photogenerated electrons thanks to the built-in electric field at the heterojunction, which inhibits the recombination of the photogenerated charge carriers, and thus further improves the photocatalytic degradation rate.

### 4.3. Polypropylene (PP)

**Figure 12:**
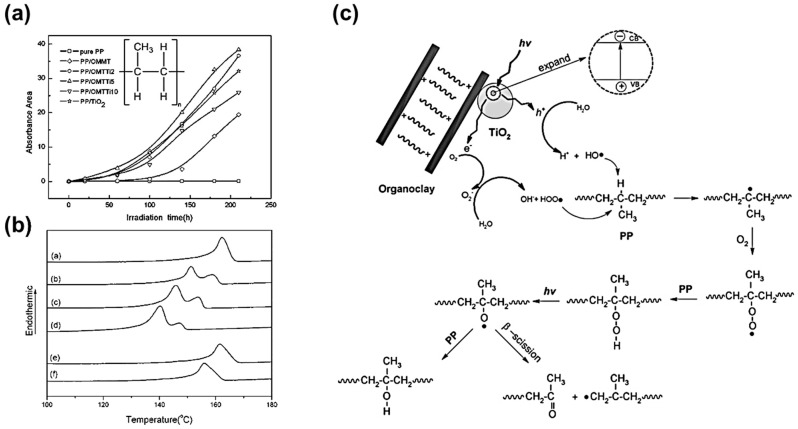
Photodegradation of PP. (**a**) Irradiation time dependent carbonyl band area of composite of pure PP, PP/OMMT, PP/OMTTi2, PP/OMTTi5, PP/OMTTi10, and PP/TiO_2_. Inset: Chemical formula of PP. (**b**) Melting endotherms of PP/OMTTi5 film irradiated for 0 h (curve a), 80 h (curve b), 170 h (curve c), 300 h (curve d), as well as that of pure PP film irradiated for 0 h (curve e) and 300 h (curve f). (**c**) Proposed mechanism for degradation of PP/TiO_2_-OMT composite [68]; reprinted from [68] with permission from John Wiley and Sons.

TiO_2_ with mixed crystalline forms (anatase and rutile) exhibits the highest activity for photodegradation of PP [(C_3_H_6_)_n_, as depicted in the inset of Figure 12a] with high consumption of oxygen [67]. Furthermore, it was found that the particle size of TiO_2_ plays an important role, and extra fine TiO_2_ is more favourable than large particles for photocatalytic degradation. Similar to the other polymers, modified TiO_2_ photocatalysts enhance the photocatalytic degradation efficiency. Meng et al. [68] investigated TiO_2_ immobilized organoclay (TiO_2_-OMT) photocatalysts. Samples with increasing mass ratio of TiO_2_-OMT photocatalyst in the clay from 2, 5, and 10 mmol Ti/g clay were prepared as PP/OMTTi2, PP/OMTTi5, and PP/OMTTi10, respectively. Pure PP, PP/OMT, and PP/TiO_2_ composites as control samples were prepared for comparison. Apparently, PP/OMTTi5 shows the highest degradation rate as reflected by the largest carbonyl band absorbance area, as depicted in Figure 12a. Differential scanning calorimetry (DSC) thermal analysis shows that PP/OMTTi5 has double endothermic melting peaks after irradiation over 80 h, as presented in Figure 12b. This observation was attributed to the decreased crystallization of PP during photodegradation, which caused the metastable crystal phase to appear as the lower DSC peak. The quantitative molecule weight characterized by gel permeation chromatography indicates a 2-order-of-magnitude reduction of molecular weight of PP after 300-h of irradiation. The photogenerated electrons and holes in TiO_2_ can react with the absorbed H_2_O or O_2_ to produce various reactive oxygen species (ROSs) including $O_{2}^{-}$, ${\mathrm{HOO}}^{•}\;$, ${\mathrm{HO}}^{•}$, etc. These ROSs capture hydrogen atoms in PP polymer chains and then generate $–{\mathrm{CH}}_{2}\left({\mathrm{CH}}_{3}\right)C^{•}–$ macro molecule radicals, followed by chain scission, as detailed in Figure 12c.

**Figure 13:**
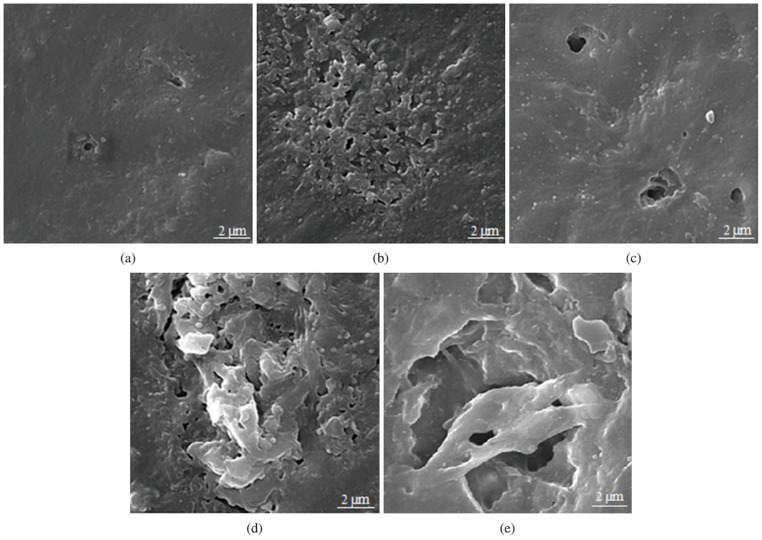
SEM images of PP nano-composite samples after 500 h of UV irradiation. (**a**) Pure PP, (**b**) PP5Ti, (**c**) PP10Ti, (**d**) PP30Ti, and (**e**) PPDTi [69].

Interestingly, carbon coating on TiO_2_ increases the photocatalytic activity compared to pure PP but not PP-TiO_2_ composite film [69]. Three types of samples were prepared by mixing carbon-coated TiO_2_ and PP with the decreasing carbon content from PP5Ti (30.6 wt%), PP10Ti (11.6 wt%), PP30Ti (2.7 wt%) to PPDTi (0 wt.%), with reference to pure PP. Direct evidence from scanning electron microscopy (SEM) images are shown in Figure 13, where the morphology of the degraded samples are presented. One can see that, after 500-h of illumination, PP30Ti and PPDTi show a very rough surface with very large cavities. In contrast, the rest of the samples show a much smoother surface, suggesting mild degradation. The authors attributed the decreased efficiency to the carbon-coated layer decreasing the amount of UV light reaching the surface of the particles, and in turn reducing the hydroxyl groups on the surface of TiO_2_. These observations thus emphasize the importance of TiO_2_-PP interface for PP photodegradation.

**Figure 14:**
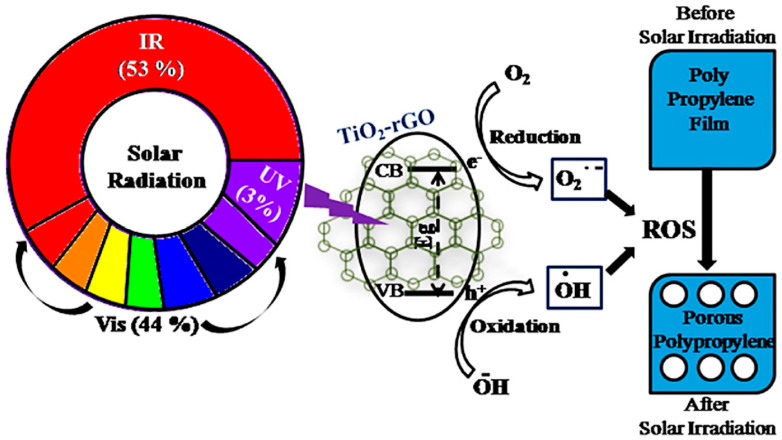
Schematic illustration of photocatalytic degradation of polypropylene by TiO_2_-rGO nanocomposite [70]; reprinted from [70] with permission from Elsevier.

Compared with the abovementioned photocatalysts including TiO_2_-OMT and carbon-coated TiO_2_, the reduced graphene oxide (rGO) coated TiO_2_ (TiO_2_-rGO) is the best for photocatalytic degradation thanks to the introduction of rGO, as illustrated in Figure 14 [70]. Briefly, upon illumination, the photogenerated electrons and holes in TiO_2_ cause reduction and oxidation reactions, respectively, leading to the formation of ROSs. The generated ROSs attack the C-H bonds in PP and generate PP macro radicals for further chain scission. The high rate of degradation was attributed to (1) rGO extending the absorption range of TiO_2_ to a visible region due to the presence of Ti-O-C bond; (2) rGO acting as a good electron acceptor thanks to its 2D π-conjugation, facilitating electron-hole separation, and thus decreasing the recombination rate; and (3) rGO offering more adsorption sites and catalytic sites.

### 4.4. Polyethylene (PE)

**Figure 15:**
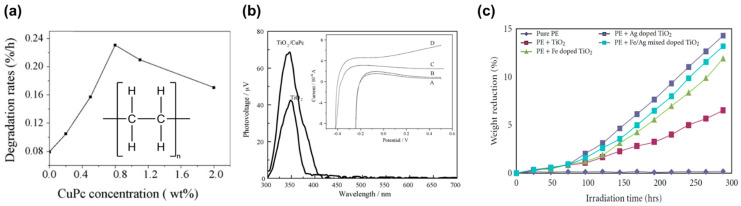
Photodegradation of PE. (**a**) CuPc concentration wt% dependent degradation rate. Inset: Chemical formula of PE. (**b**) Surface photovoltage spectroscopy spectra of TiO_2_ and TiO_2_/CuPc photocatalyst. Inset: The I-V curves of different photocatalysts, i.e., TiO_2_/CuPc without irradiation (curve A), TiO_2_/CuPc under visible irradiation (curve B), TiO_2_ under UV irradiation (curve C), and TiO_2_/CuPc under UV radiation (curve D) [72]; reprinted from [72] with permission from Elsevier. (**c**) Effect of simulated sunlight on the photocatalytic degradation of PE film [74].

The photocatalytic degradation rate of PE (see inset of Figure 15a for molecular structure) is found not linearly proportional to the TiO_2_ concentration [71]. The experiment using TiO_2_/CuPc as photocatalyst to degrade the PE plastic has been conducted, and the results showed that PE-TiO_2_/CuPc has a higher photocatalytic degradation rate than PE-TiO_2_ [72]. However, there was an optimal concentration of CuPc, i.e., 0.8 wt% for the highest photocatalytic degradation rate, probably caused by the trade-off between absorption in unit area and light penetration depth, as displayed in Figure 15a. The author furthers characterized the photovoltage using surface photovoltage spectroscopy (SPS), as depicted in Figure 15b. In the range of 300–400 nm, TiO_2_/CuPc shows a much higher photovoltage, suggesting a higher charge separation efficiency and longer excitations lifetime than that of TiO_2_. Moreover, the TiO_2_/CuPc sample displays a broader range of photo response. However, there is no response in the visible region, indicating that there is no charge transfer between TiO_2_ and CuPc in the visible range. The current-potential curve further shows that visible light illumination did not make a difference in photocurrent on TiO_2_/CuPc, as shown in the inset of Figure 15b. However, the photocurrent of TiO_2_/CuPc is much larger than that of TiO_2_. Furthermore, due to the different molecular structures, it is found that the degradation of PS-TiO_2_/CuPc was slower than PE-TiO_2_/CuPc.

Fa et al. studied the photocatalytic degradation of the polyethylene-oxidized polyethylene wax-TiO_2_ (PE-OPW-TiO_2_) [73]. The presence of OPW helps improve the interaction between PE and modified TiO_2_ particles, further improving the degradation efficiency. Photodegradation of doped TiO_2_ composite films including Fe/Ag dually doped TiO_2_, Ag-doped TiO_2_, and Fe doped TiO_2_ were investigated [74]. Overall, the degradation of doped TiO_2_ was greater than that of the undoped TiO_2_, and Fe/Ag dually doped TiO_2_ shows the highest degradation rate under UV light among all, as shown in Figure 15c.

**Figure 16:**
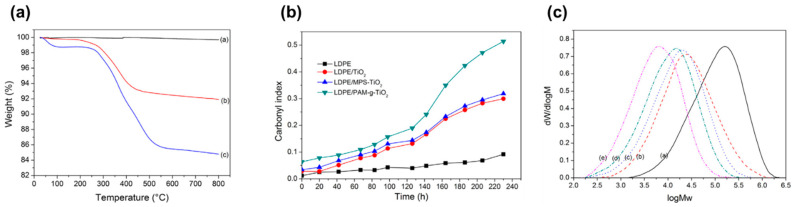
Photodegradation of PE-PAM-g-TiO_2_ film. (**a**) Thermogravimetric analysis of TiO_2_ (curve a), MPS-TiO_2_ (curve b), and PAM-g-TiO_2_ (curve c). (**b**) The carbonyl index of various samples under UV irradiation. (**c**) Molecular weight distribution of films LDPE (curve a), LDPE-UV (curve b), LDPE/TiO_2_-UV (curve c), LDPE/MPS-TiO_2_-UV (curve d), and LDPE/PAM-g-TiO_2_-UV (curve e) with irradiation time of 520 h [77]; reprinted from [77] with permission from Elsevier.

Moreover, the photocatalytic activity of PE-TiO_2_ can be improved using modified TiO_2_ photocatalysts such as multiwalled carbon nanotube (MWCNT)-TiO_2_ composite (TiO_2_-MWCNTs) [75], polypyrrole(PPy)-TiO_2_ composite (PPy/TiO_2_) [76], and polyacrylamide grafted TiO_2_ (PAM-g-TiO_2_) [77]. PAM absorbs moisture from the atmosphere due to its high hydrophilicity, which results in the weight loss of PAM-g-TiO_2_ sample before 100 °C on the thermogravimetric analysis curve, as shown in Figure 16a. The absorbed moistures produced more hydroxyl radicals which would accelerate the photodegradation. The carbonyl index measurement in Figure 16b increases with the increasing UV exposure duration due to the initiation of chain scission caused by photocatalytic oxidation, which produces carbonyl compounds with low molecular weights (e.g., ester, acid, and aldehyde). The degraded PAM also generates amide and acid that promote the degradation of LDPE. As displayed in Figure 16c, the molecular weight (M_w_) distribution curve peak shifts to the lower value from LDPE towards the LDPE/PAM-g-TiO_2_-UV sample, indicating that the PAM-g-TiO_2_ has the highest activity towards LDPE degradation under UV irradiation.

## 5. Conclusions and Outlook

In recent years, plastic pollution has increasingly shown a negative impact on our environment and health. The recycling rate of plastic waste is very low (~10%) mainly caused by the contamination of plastic waste by other solid waste, as well as the fact that the mixing nature of plastic waste consists of various polymers. Additionally, the widespread plastic waste makes collection and transportation necessary for centralized recycling technology such as mechanical and thermal catalytic recycling. Thus, a key to increase the recycling rate of plastic waste, and thus less disposal of plastic waste to landfill, is to develop a decentralized degradation process that could leverage renewable energies as the driving force. To this end, photodegradation is perfect as sunlight is known to be an effective energy source to degrade plastic. Therefore, we present this short review to recap the photocatalytic degradation mechanism and to discuss the research efforts on photodegradation of various plastic polymers, aiming to inspire more research ideas to address this urgent challenge of plastic waste. Despite the advantages of photocatalysis and extensive efforts devoted to plastic photodegradation to date, there are still several major challenges as follows:

(1) Photocatalytic degradation mechanism. Decomposition of a large plastic polymer to small molecules is mechanistically complicated. There could be a dozen or more different reaction pathways. How to identify and then to control the reaction pathway is a paramount challenge. Some in situ/operando characterizations could be useful, such as Raman spectroscopy, photoluminescence spectroscopy, and high-resolution soft X-ray absorption spectroscopy. It is urgent to develop a suitable and effective characterization tool/method for in situ/operando monitoring of the degradation process. Theoretical investigation including first-principle modelling and microkinetic modelling complementary to the in situ/operando studies could be powerful for reaction pathways investigation.
(2) Contamination-tolerant degradation technology. Most of the plastic wastes are generated in a widespread manner and contaminated by various other wastes such as food wastes, wood waste, and chemical waste. Though the photocatalytic process shows good tolerance to these contaminations, it is a surface reaction. Thus, its efficiency would be low if light penetration is blocked by the non-transparent contaminations. Thus, a photocatalyst that could degrade these contaminations should be developed and used together with the plastic degradation photocatalyst.
(3) Multifunctional photocatalyst. Very often, a plastic product consists of various plastic components, e.g., electronic plastic may contain polyimide, ABS, etc. Therefore, a multifunctional photocatalyst that consists of individual elements working for a specific plastic could be very useful for addressing the plastic waste issue. Moreover, the selectivities of these catalysts are crucial from an economical perspective. Most of the studied catalyst generates CO_2_ as the major product. Though less harmful than plastic waste, CO_2_ is also a serious environmental issue to be addressed.
(4) Facile processes to introduce photocatalyst into plastic waste. Most of the current work involves dissolving plastics followed by mixing with photocatalyst to make a composite, which is then photodegraded. Considering the low economic viability of such a process, a new method to introduce photocatalyst into plastic waste is needed. For instance, dispersion of photocatalyst particles that can stick on bulk plastic waste could be attractive as it is facile and cost-effective. However, an intensive research effort may be necessary for developing such a photocatalyst.

## Figures and Tables

**Table 1:** SPI number for different types of plastic and the recovery rate from the total solid plastic waste [1].

SPI Number	Full Name	Chemical Structure	Uses	Currently Recyclable?	Recovery Rate (%)
1	Polyethylene terephthalate	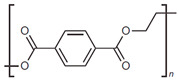 PETE/PET	Disposable bottles for drinks, medicines, and many other consumer products	Yes	19.5
2	High-density polyethylene	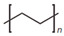 HDPE	More durable containers, such as those for detergent, bleach, shampoo or motor oil	Yes	10
3	Polyvinyl chloride	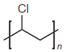 PVC	Piping, cables, garden furniture, fencing and carpet backing	No	0
4	Low-density polyethylene	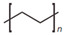 LDPE	Plastic bags, wrapping films, trays and computer components	Mostly no	5
5	Polypropylene	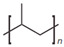 PP	Bottle caps, reusable food containers and car parts	Sometimes	1
6	Polystyrene	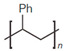 PS	Plastic utensils, packaging peanuts and Styrofoam (EPS)	Sometimes	1
7	Other: for example, polycarbonate and polymethyl methacrylate	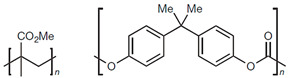 PMMA PC	Multilayer barrier films, toothbrushes, some food containers, CDs, and DVDs	No	Varies
